# “Get with the Guidelines Heart Failure Risk Score” for mortality prediction in patients undergoing MitraClip

**DOI:** 10.1007/s00392-021-01804-3

**Published:** 2021-01-31

**Authors:** Christos Iliadis, Maximilian Spieker, Refik Kavsur, Clemens Metze, Martin Hellmich, Patrick Horn, Ralf Westenfeld, Vedat Tiyerili, Marc Ulrich Becher, Malte Kelm, Georg Nickenig, Stephan Baldus, Roman Pfister

**Affiliations:** 1 Department of Cardiology, Angiology, Pneumology and Medical Intensive Care, Heart Center of the University of Cologne, University of Cologne, Faculty of Medicine and University Hospital Cologne, Kerpener Str. 62, 50937 Cologne, Germany; 2 Department of Cardiology, Pulmonology and Vascular Medicine, Heinrich-Heine University Düsseldorf, Medical Faculty, Düsseldorf, Germany; 3 Department of Cardiology, Angiology, Pneumology and Medical Intensive Care, University Hospital Bonn, Bonn, Germany; 4 Institute of Medical Statistics and Computational Biology, Faculty of Medicine and University Hospital Cologne, University of Cologne, Cologne, Germany

**Keywords:** MitraClip, Mortality, Get with the guidelines heart failure risk score, Heart failure

## Abstract

**Background:**

Reliable risk scores in patients undergoing transcatheter edge-to-edge mitral valve repair (TMVR) are lacking. Heart failure is common in these patients, and risk scores derived from heart failure populations might help stratify TMVR patients.

**Methods:**

Consecutive patients from three Heart Centers undergoing TMVR were enrolled to investigate the association of the “Get with the Guidelines Heart Failure Risk Score” (comprising the variables systolic blood pressure, urea nitrogen, blood sodium, age, heart rate, race, history of chronic obstructive lung disease) with all-cause mortality.

**Results:**

Among 815 patients with available data 177 patients died during a median follow-up time of 365 days. Estimated 1-year mortality by quartiles of the score (0–37; 38–42, 43–46 and more than 46 points) was 6%, 10%, 23% and 30%, respectively (*p* < 0.001), with good concordance between observed and predicted mortality rates (goodness of fit test *p* = 0.46). Every increase of one score point was associated with a 9% increase in the hazard of mortality (95% CI 1.06–1.11%, *p* < 0.001). The score was associated with long-term mortality independently of left ventricular ejection fraction, NYHA class and NTproBNP, and was equally predictive in primary and secondary mitral regurgitation.

**Conclusion:**

The “Get with the Guidelines Heart Failure Risk Score” showed a strong association with mortality in patients undergoing TMVR with additive information beyond traditional risk factors. Given the routinely available variables included in this score, application is easy and broadly possible.

**Graphic abstract:**

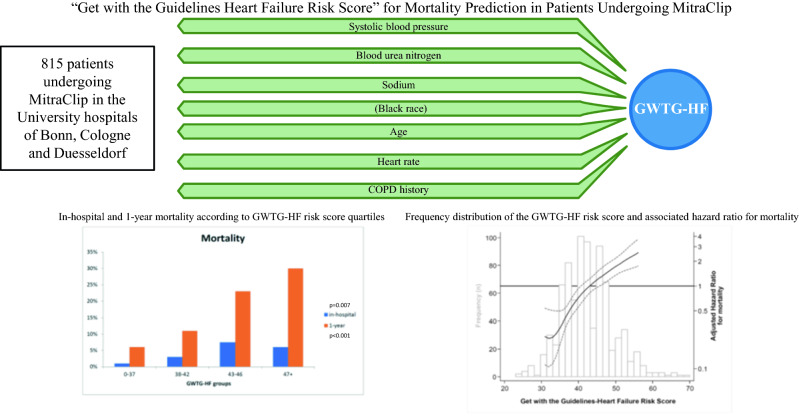

**Supplementary Information:**

The online version contains supplementary material available at 10.1007/s00392-021-01804-3.

## Introduction

Transcatheter mitral valve repair (TMVR) with MitraClip is a recommended treatment for selected patients with primary and secondary mitral regurgitation (MR) and high or prohibitive surgical risk [1]. TMVR has shown an excellent safety profile and high technical success [2, 3]. However, outcome after TMVR is impaired due to severe cardiac and non-cardiac morbidities [4]. Risk stratification in these patients is important for informed decision making of physicians, patients and relatives. This might for instance refer to decisions on a conservative or palliative strategy or advanced heart failure interventions such as assist devices in patients with a futile risk profile.

So far, reliable risk stratification in patients undergoing TMVR is lacking. Scores derived from surgical patients predicting peri-operative mortality like logistic Euroscore, Euroscore II and Society of Thoracic Surgeons score, show weak stratification in TMVR patients [5]. Other risk models proposed exclusively for TMVR patients were derived from small patient cohorts [6]. Although several studies have reported individual risk factors for adverse outcome after TMVR, many of these are single-center studies [7, 8], used variables not commonly available in routine [9], or predictive variables were inconsistent across studies [10, 11].

The majority of patients undergoing TMVR have secondary MR with underlying left ventricular (LV) dysfunction and heart failure (HF). Furthermore, also patients undergoing TMVR for primary MR often have a history of symptomatic HF and impaired LV function [12]. We hypothesized that a risk score derived from HF patients might be associated with outcome in patients undergoing TMVR. Aim of this study was to examine the association of the “Get-with-the-Guidelines Heart Failure (GWTG-HF) risk score” with all-cause mortality and validate the score in a large multi-center cohort of patients undergoing TMVR. The GWTG-HF score is so far only validated for prediction of mortality in hospitalized and pre-discharge patients with HF [13, 14].

## Methods

**Fig. 1:**
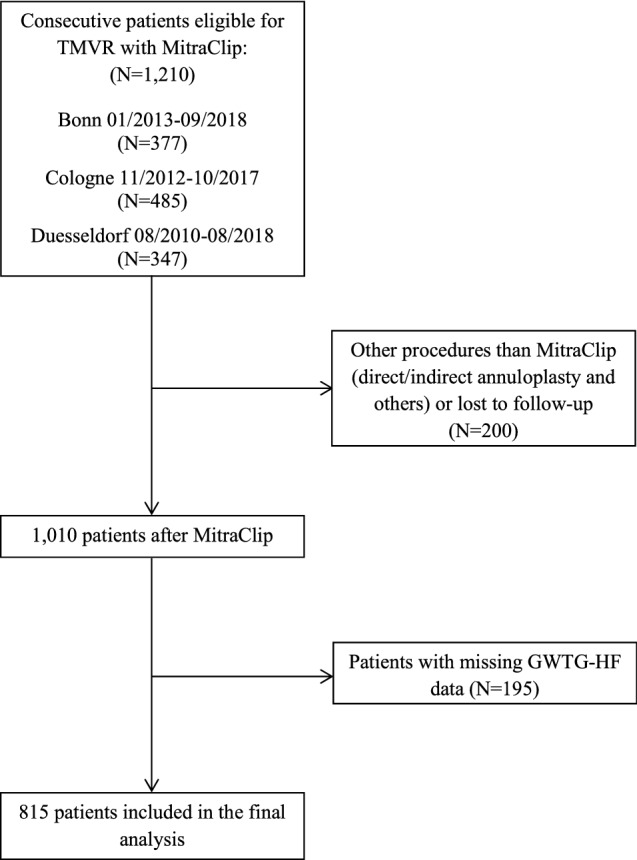
Study flow chart

Data of 1010 patients who underwent TMVR with the MitraClip system in the Heart Failure Network Rhineland (University hospitals of Bonn, Cologne and Duesseldorf) between 2010 and 2018 were retrospectively analyzed for parameters of the GWTG-HF score. The study received the proper ethical oversight and the data collection has been previously approved by the ethics committee of the University of Bonn, Cologne and Duesseldorf, respectively. The three tertiary care hospitals are high-volume referral centers for valvular heart disease providing the whole spectrum of surgical and catheter based mitral valve therapies. Briefly, all patients were discussed by a Heart Team, including at least one interventional cardiologist, one non-interventional cardiologist and one cardiac surgeon, and a decision on interventional treatment approach was made based on surgical risk, MR etiology, morphological suitability and other relevant patient characteristics. Only patients with deployment of at least one MitraClip device, complete follow-up data on vital status and complete data of the GWTG-HF score were enrolled in this analysis (Fig. 1). Baseline characteristics were assessed before the TMVR procedure and collected from records. Importantly, the Heart Failure Network Rhineland is independent from industry.

### GWTG-HF risk score

The GWTG-HF score [13] is a mortality predictive model for patients hospitalized with HF that has been validated independently [15]. It uses commonly available clinical variables to predict in-hospital mortality as a tool for mortality risk stratification that is applicable to a broad spectrum of HF patients, including those with preserved LV systolic function [16]. The GWTG-HF score can easily be calculated from systolic blood pressure, blood urea nitrogen, blood sodium, age, heart rate, race, and history of chronic obstructive pulmonary disease using an online calculator (www.mdcalc.com/gwtg-heart-failure-risk-score). Scores range from 0 to 100, with scores 0–33 having < 1% probability of death to scores over 79 having > 50% probability of death, with a continuous increase of death risk associated with increases in score.

### Endpoint

The clinical course was monitored by institutional follow-up examinations, phone calls with the referring cardiologists and the patients’ primary physicians or the patients themselves. The endpoint was all-cause mortality, given the fact that GTWTG-HF score has been proposed as a mortality prediction model.

### Statistical analysis

The GWTG-HF score showed a continuous risk increase with increasing score values in previous studies, without evidence for a threshold effect. Hence, we used quartiles of the GWTG-HF score to examine the shape of association with mortality after TMVR and baseline characteristics. Patient characteristics were compared between quartiles using the chi-square test (or Fisher’s exact test if the expected count was less than five per cell) for categorical variables. All continuous variables were distributed non-normally according to the Kolmogorov–Smirnov test and were examined by Kruskal–Wallis test for analysis of differences in distribution. Percentages were reported to describe categorical variables and median (interquartile range) was reported for continuous variables. The Kaplan–Meier method was used for presenting the event-free survival. The observation time on the Kaplan–Meier plot was truncated at 75% of the total follow-up time. Log-rank test was used to examine differences across groups. Subgroup analysis was performed by etiology of MR (primary or secondary) and LV-EF (≥ 50% or < 50%). Due to low numbers in patients with primary MR, tertiles of the GWTG-HF score were used instead of quartiles. Cox proportional hazard models were used to assess the contribution of the GWTG-HF score, individual components of the GWTG-HF score and other risk factors for mortality. The proportional hazards assumption was tested on the basis of Schoenfeld residuals after fitting a Cox model. Hazard ratio and 95% confidence interval are presented. To assess the continuous association between GWTG-HF score and mortality, we generated restricted cubic splines with knots at the 5th, 25th, 75th and 95th percentiles of the GWTG-HF score (33, 37, 46 and 55 points), centred at the median (42 points), and the plot was truncated at the 2.5th and 97.5th percentiles. Score discrimination was assessed using Harrell’s c-statistic. The calibration of the score’s performance was analyzed using a goodness-of-fit test proposed by Gronnesby and Borgan for Cox proportional hazard models and using Arjas like plots comparing counts of observed and expected events at each event time. For all analyses, a *p* value of < 0.05 was considered to be statistically significant. Analyses were performed using SPSS Statistics 25 (IBM, Armonk, NY, USA) and Stata/SE 12.1 (StataCorp LP, College Station, TX, USA).

## Results

### Baseline characteristics

A total of 815 patients who underwent TMVR with MitraClip were included into the analysis. Median age was 78 (73–83) years, 43% were female. Median NTproBNP was 2529 (1392–5499) ng/l and left ventricular ejection fraction (LV-EF) was severely reduced (< 30%) in 185 (22.8%) patients. Median estimated glomerular filtration rate was 48 (35–64) ml/min/1.73 m^2^. Secondary MR was present in 509 (62.5%) patients. The median Euroscore was 19.2 (10.4–31.3). The study population differed significantly from the 195 patients excluded from the analysis. The study population had more women, more secondary etiology of MR, previous strokes, diabetes mellitus, previous bypass surgery, higher Euroscore levels, and lower rates of transitory ischemic attacks and hypertension compared to the patients excluded from the analysis (Supplementary Table 1).

**Table 1:** Baseline characteristics according to GWTG-HF score quartiles

	GWTG-HF 0–37 (*N* = 210)	GWTG-HF 38–42 (*N* = 218)	GWTG-HF 43–46 (*N* = 186)	GWTG-HF 47+ (*N* = 201)	*p* value
Median GWTG-HF score (IQR)	35 (33–37)	41 (39–41)	45 (43–45)	50 (48–54)	< 0.001
Age (years)	76 (67–81)	79 (75–83)	79 (74–83)	79 (74–84)	< 0.001
Systolic blood pressure (mmHg)	140 (130–150)	125 (120–135)	115 (107–125)	105 (96–115)	< 0.001
BUN (mg/dl)	18 (15–24)	22 (17–31)	28 (23–39)	44 (32–62)	< 0.001
Sodium (mmol/l)	140 (139–142)	140 (138–141)	139 (137–141)	138 (135–140)	< 0.001
Heart rate (bpm)	70 (64–76)	70 (62–80)	70 (64–80)	76 (68–90)	< 0.001
Black race	0 (0%)	0 (0%)	1 (0.5%)	0 (0%)	Not analyzed
COPD	30 (14.3%)	45 (20.6%)	42 (22.6%)	46 (22.9%)	0.10
Female, *n* (%)	97 (46.2%)	105 (48.2%)	75 (40.3%)	74 (36.8%)	0.07
NYHA functional class	(*N* = 210)	(*N* = 217)^a^	(*N* = 186)	(*N* = 201)	0.001
I	4 (1.9%)	2 (0.9%)	3 (1.6%)	1 (0.5%)	
II	40 (19%)	22 (10.1%)	16 (8.6%)	13 (6.5%)	
III	144 (68.6%)	155 (71.4%)	132 (71%)	141 (70.1%)	
IV	22 (10.5%)	38 (17.5%)	35 (18.8%)	46 (22.9%)	
Left ventricular ejection fraction	(*N* = 209)^a^	(*N* = 218)	(*N* = 185)^a^	(*N* = 200)^a^	0.06
< 30%, *n* (%)	37 (17.7%)	43 (19.7%)	46 (24.9%)	59 (29.5%)	
30–50%, *n* (%)	68 (32.5%)	77 (35.3%)	66 (35.7%)	64 (32%)	
≥ 50%, *n* (%)	104 (49.8%)	98 (45%)	73 (39.5%)	77 (38.5%)	
Secondary etiology of MR, *n* (%)	136 (64.8%)	135 (61.9%)	113 (60.8%)	125 (62.2%)	0.86
Peripheral arterial disease, *n* (%)	16 (7.6%) (*N* = 210)	33 (15.2%) (*N* = 217)^a^	25 (13.4%) (*N* = 186)	41 (20.4%) (*N* = 201)	0.003
Previous myocardial infarction, *n* (%)	49 (23.4%) (*N* = 209)^a^	68 (31.3%) (*N* = 217)^a^	57 (30.8%) (*N* = 185)^a^	59 (29.4%) (*N* = 201)	0.26
Previous TIA, *n* (%)	0 (0%)	2 (0.9%)	1 (0.5%)	5 (2.5%)	0.24
Previous stroke, *n* (%)	27 (12.9%)	22 (10.1%)	26 (14%)	26 (12.9%)	0.24
Atrial fibrillation, *n* (%)	122 (58.7%) (*N* = 208)^a^	140 (64.5%) (*N* = 217)^a^	117 (63.6%) (*N* = 184)^a^	132 (66%) (*N* = 200)^a^	0.44
Hypertension, *n* (%)	180 (85.7%)	178 (81.7%)	149 (80.1%)	163 (81.1%)	0.47
Diabetes mellitus, *n* (%)	61 (29%)	63 (28.9%)	61 (32.8%)	70 (34.8%)	0.49
Estimated GFR (ml/min/1.73 m^2^)	62 (46–73) (Ν = 208)^a^	54 (41–66) (Ν = 215)^a^	44 (33–55) (Ν = 184)^a^	35 (24–45) (Ν = 199)^a^	< 0.001
Previous CABG, *n* (%)	85 (40.5%)	98 (45%)	77 (41.4%)	84 (41.8%)	0.8
NTproBNP (ng/l)	1623 (874–3354) (Ν = 172)^a^	2340 (1412–4293) (Ν = 163)^a^	2941 (1629–5966) (Ν = 157)^a^	4526 (2276–8793) (Ν = 145)^a^	< 0.001
Euroscore (%)	15.4 (9.6–28.2) (Ν = 202)^a^	19.5 (10.3–29.3) (Ν = 211)^a^	19.3 (10.9–33.3) (Ν = 181)^a^	22.7 (11.9–35.1) (Ν = 197)^a^	0.002
Heart failure decompensation within 12 months, *n* (%)	84 (40%) (*N* = 208)^a^	110 (50.5%) (*N* = 218)	106 (57%) (*N* = 183)^a^	122 (60.7%) (*N* = 199)^a^	< 0.001
Heart failure medical therapy	(*N* = 210)	(*N* = 218)	(*N* = 186)	(*N* = 201)	
ACEI/ARB/ARNI, *n* (%)	175 (83.3%)	165 (75.7%)	141 (75.8%)	152 (75.6%)	0.16
MRA, *n* (%)	81 (38.6%)	79 (36.2%)	99 (53.2%)	107 (53.2%)	< 0.001
Betablocker, *n* (%)	186 (88.6%)	197 (90.4%)	158 (84.9%)	182 (90.5%)	0.27
Digitalis glycosides, *n* (%)	21 (10%)	32 (14.7%)	16 (8.6%)	15 (7.5%)	0.08
Diuretics, *n* (%)	183 (87.1%)	194 (89%)	172 (92.5%)	185 (92%)	0.23
Deceased, *n* (%)	19 (9%)	34 (16%)	47 (25%)	77 (38%)	< 0.001

Displayed are median and interquartile range or numbers and percentages; *p* values: Kruskal–Wallis test or chi-square test/Fisher’s Exact test

^a^Only available in *N* of the patients

### GWTG-HF score in patients undergoing TMVR

The median GWTG-HF score was 42 (37–46). Baseline characteristics according to quartiles of the GWTG-HF score (score range of quartiles: 0–37, 38–42, 43–46 and 47 +) are summarized in Table 1. All individual score components were significantly different across quartiles. NYHA functional class significantly increased, as did NTproBNP, the Euroscore, and rate of peripheral artery disease, previous decompensation and mineralocorticoid-receptor antagonist use, whereas renal function significantly decreased with higher quartiles of the score. LV-EF groups did not significantly differ across the GWTG-HF score quartiles. There was no difference in the frequency of MR etiology between the four groups.

### GWTG-HF score and clinical outcome

**Fig. 2:**
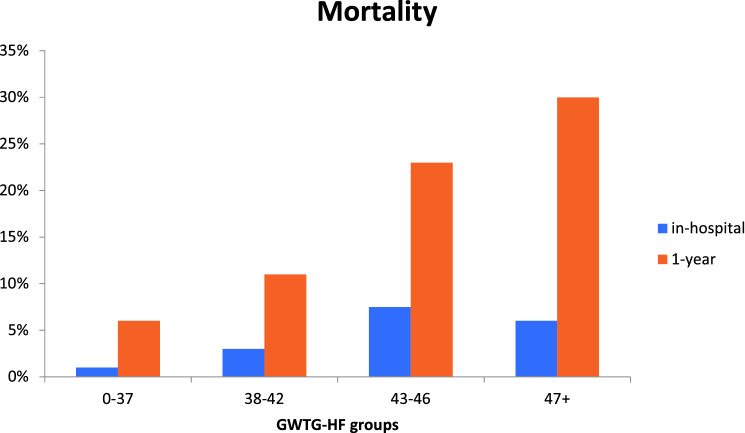
In-hospital and 1-year mortality according to GWTG-HF score quartiles

The median follow-up of the study population was 365 (365–546) days. During the follow-up period, 177 (21.7%) patients died. In-hospital mortality across quartiles was observed in 3 (1%), 6 (3%), 14 (7.5%) and 12 (6%) patients, respectively (*p* = 0.007). Mortality during the first year after the procedure was observed in 12 (6%), 24 (11%), 42 (23%) and 60 (30%) patients, respectively (*p* < 0.001, Fig. 2).

**Fig. 3:**
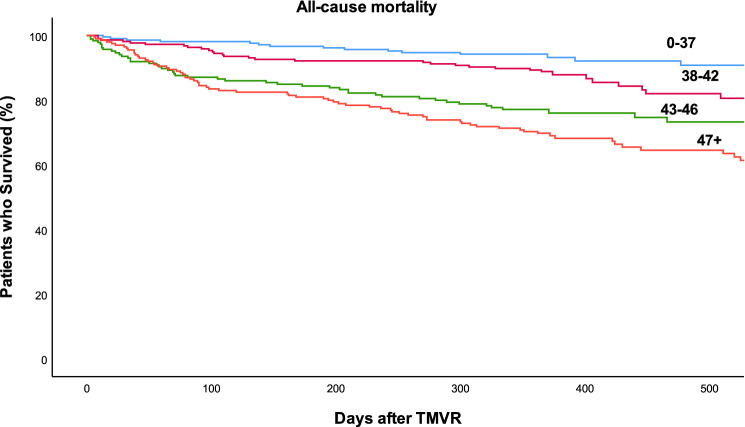
Kaplan–Meier plot for mortality by GWTG-HF score quartiles

**Fig. 4:**
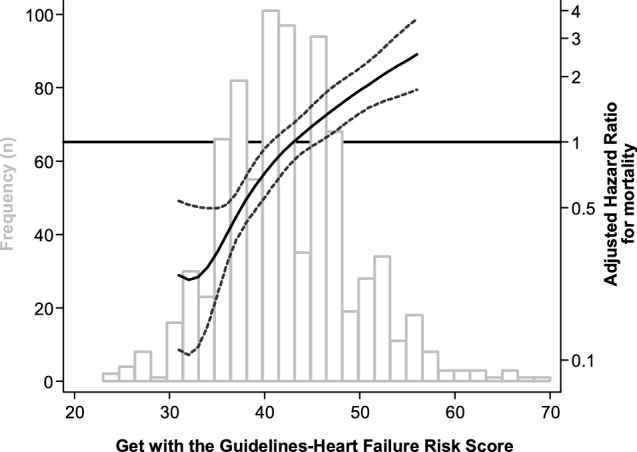
Frequency distribution of the GWTG-HF score (left y axis, grey bars) and associated hazard ratio of mortality using cubic spline analysis (right *y* axis, black line, with 95% confidence interval dotted lines)

Kaplan–Meier analysis and log-rank test showed a significantly reduced survival of patients by increasing quartiles of the GWTG-HF score (*p* < 0.001, Fig. 3). Compared to patients of the bottom score quartile, patients of the second, third and fourth quartile had a hazard ratio of mortality of 1.78 (95% CI 1.02–3.12%), 3.17 (95% CI 1.86–5.41) and 4.68 (95% CI 2.83–7.73), respectively. Every increase in one GWTG-HF score point was associated with a 9% higher hazard (95% CI 6–11%, *p* < 0.001) of mortality. Restricted cubic spine analysis showed a continuous increase of mortality risk across the range of the GWTG-HF score from 33 to 57 points (Fig. 4). Risk discrimination of the GWTG-HF score for all-cause mortality was moderate with a c-statistic of 0.68. The calibration of the score was examined by comparing observed vs. predicted numbers of death events by quartiles of the score. As shown in Supplementary Table 2 and Supplementary Fig. 1, there was excellent concordance between observed and model-predicted mortality rates across quartiles of the GWTG-HF score as determined by the Score test for goodness of fit (*p* = 0.46).

The association of the GWTG-HF score with mortality was attenuated but remained significant when adjusting for other measures of heart failure severity such as NYHA class, left-ventricular ejection fraction, NT-pro-BNP and previous hospitalization of heart failure (HR 1.06, 95% CI 1.03–1.09, *p* < 0.001). When analyzing the prognostic impact of individual GWTG-HF score components, blood urea nitrogen, systolic blood pressure and heart rate showed the strongest association with mortality (Table 2).

**Table 2:** Analysis of individual GWTG-HF score components

	Hazard ratio	95% confidence interval	*p* value
BUN (per IQR: 21 mg/dl)	1.55	1.36–1.77	< 0.001
Systolic blood pressure (per IQR: 25 mmHg)	0.66	0.55–0.78	< 0.001
Sodium (per IQR: 4 mmol/l)	0.80	0.69–0.93	0.004
Age (per IQR: 10 years)	1.24	1.03–1.49	0.025
Heart rate (per IQR: 16 beats/min)	1.33	1.14–1.55	< 0.001
Black race	Not analyzed		
COPD	1.39	0.98–1.97	0.066

Cox regression analysis for all-cause mortality

*ACEI* angiotensin converting enzyme inhibitor, *ARB* angiotensin-receptor blocker, *ARNI* angiotensin-receptor blocker—neprilysin inhibitor, *bpm* beats per minute, *BUN* blood urea nitrogen, *CABG* coronary artery bypass grafting, *COPD* chronic obstructive pulmonary disease, *eGFR* estimated glomerular filtration rate, *GWTG-HF* get-with-the-guidelines heart failure, *IQR* interquartile range, *ln NTproBNP* base-e logarithm N-terminal pro-brain natriuretic peptide, *LV-EF* left ventricular ejection fraction, *MR* mitral regurgitation, *MRA* mineralcorticoid-receptor antagonist, *NTproBNP* N-terminal pro-brain natriuretic peptide, *NYHA* New York Heart Association, *TIA* transitory ischemic attack

Subgroup analysis stratified by MR etiology and EF impairment revealed a similar association between all-cause mortality and GWTG-HF score as in the total population (Supplementary Figs. 2–5).

When comparing the GWTG-HF score with the Euroscore, the discriminatory performance of the GWTG-HF score for mortality was higher according to ROC analysis (Supplementary Fig. 6). The area under the ROC curve for 30-day mortality was 0.65 in GWTG-HF vs. 0.62 for Euroscore, with a p-value of 0.65 for comparison. The area under the ROC curve for 1-year mortality was 0.71 in GWTG-HF vs. 0.57, with a p-value of < 0.001 for comparison. After performing a bivariate analysis of the two score systems for mortality, the association of GWTG-HF with mortality was virtually the same, resulting in an 8% higher hazard (95% CI 6–10%, *p* < 0.001) with every increase in one GWTG-HF score point (Supplementary Table 3).

## Discussion

In the present study we investigated the performance of the GWTG-HF score for mortality prediction in patients undergoing TMVR with the MitraClip system. First, the GWTG-HF score was significantly associated with all-cause mortality, with a continuously increasing risk of mortality across the range of the score from 33 to 57 points and a 9% risk increase for every increase in one score point. The concordance between score predicted and observed mortality rates was excellent. Second, the association of the GWTG-HF score with mortality was independent of other HF markers such as NYHA class and NTproBNP. Third, the association of the GWTG-HF score with mortality was consistent in patients with primary and secondary MR and in patients with preserved and reduced ejection fraction.

The GWTG-HF score has initially been derived and validated in hospitalized patients with HF to predict in-hospital mortality [13, 15]. In recent studies risk prediction was not only demonstrated for in-hospital mortality but also long-term prognosis in HF patients [14, 17]. Although many patients undergoing TMVR have HF with LV dysfunction and thus might be represented by the derivation and validation cohorts of the GWTG-HF score, it was not clear whether the risk association of the score remains valid after TMVR, which has the potential to improve the disease course and attenuate mortality in these patients. Thus, we extend existing data on the risk prediction of the GWTG-HF score to HF patients undergoing TMVR, and also to patients with primary MR undergoing TMVR. Importantly, almost two thirds of our patients with primary MR had preserved LV ejection fraction.

The components comprising the GWTG-HF score showed a significant association with mortality risk, except for chronic obstructive pulmonary disease. Association with mortality in the setting of TMVR has been described before regarding renal function [18], resting heart rate [19] and higher age [20]. To our best knowledge, blood pressure and baseline sodium have not been evaluated as a risk factor in TMVR patient so far. However, in patients with HF and reduced ejection fraction both low systolic blood pressure and hyponatremia are well-established risk factors which makes our findings plausible [21].

Markers of heart failure severity such as NTproBNP, NYHA class and LV-EF are associated with mortality in TMVR [4, 11] underlining the relevance of HF as an underlying disease in most TMVR patients. Nonetheless, the GWTG-HF score showed a prognostic impact independently to these clinically important heart failure markers which is an important finding of this study and supports the additive prognostic value of the GWTG-HF score.

Major advantages of the GWTG-HF score with respect to other risk scores used in HF patients [22, 23] are the familiarity for most cardiologists, the ease of calculation using an online tool, the parsimony of variables included, and the availability of these variables in literally every patient admitted for TMVR. These are crucial reasons for a broad use of the score in clinical routine [17, 24]. The GWTG-HF score permits a continuous characterization of individual mortality risk. Still, for clinical decision-making cut-offs are usually inevitable. Such cut-offs guiding treatment decisions need to be defined in further studies. For instance, patients in the top decile of the GWTG-HF score (52 points or more) had a 1-year mortality of 50% and patients with primary MR even 59%. For these patients TMVR might be regarded futile. Of note, exclusion of patients with very advanced HF is one point under discussion to explain the divergent results of the trials COAPT and MITRA-FR in patients with secondary MR undergoing TMVR. The criterion used in COAPT to classify and exclude advanced HF was “stage D” according to the American Heart Association, which is not well defined. A score-based risk prediction might be a more objective measure to define patients without benefit from TMVR in the future.

## Strengths and limitations

A strength of this study is the large sample size and the multi-center approach which increases generalizability of our findings. However, we had only one black patient so that no conclusion can be drawn on the prognostic role of race in TMVR. Furthermore, only about 80% of patients treated at our centers with MitraClip were included in the study due to missing covariates, with evidence for the inclusion of more diseased patients. When compared to other large MitraClip registries our population showed very similar frequency of comorbidities and 1-year mortality, indicating yet good representativeness [11, 12, 25]. We had only all-cause mortality available as outcome measure. Non-fatal events such as hospital admission for HF are also important from the patient`s and physician’s perspective and might be associated with the GWTG-HF score. Indeed, in 309 patients, where data on hospitalization for HF were available, a significant 6% hazard increase was observed for each increase of a GWTG-HF score point (data not shown). The risk association with mortality is only valid for the GWTG-HF score range of 33–57 points, which was represented in our study population. This might seem a narrow range given the total score range from 0 to 100 points. However, even in classical heart failure populations up to 80% of patients have score values within the range of 33–57 points [17, 24], indicating that the GWTG-HF score will provide valid estimates for the majority of TMVR patients.

## Conclusion

The GWTG-HF score showed a strong association with short- and long-term mortality in patients undergoing TMVR, with good concordance between observed and expected mortality rates and additive information beyond traditional heart failure markers. Given the routinely available variables included in this score and the ease of calculation, a broad application is possible and might improve risk stratification in these patients.

### Clinical perspective

#### What is known?

The GWTG-HF score has been developed to predict mortality in patients hospitalized due to heart failure. It has been validated independently for in-hospital mortality and long-term prognosis.

#### What is new?

In this study we stratified 815 patients who underwent TMVR with MitraClip according to their GWTG-HF score into quartiles and evaluated the association between GWTG-HF score and clinical outcomes. The score was associated with a continuously increasing risk of mortality across the range of the score from 33 to 57 points and a good concordance between observed and expected mortality rates.

#### What is next?

The GWTG-HF score can be used for routine risk prediction in patients undergoing TMVR with MitraClip.

## Supplementary Information

Below is the link to the electronic supplementary material.

Supplementary file1 (DOCX 20 KB)

Supplementary file2 (PDF 860 KB)

## Abbreviations

GWTG-HF: Get-with-the-guidelines heart failure
HF: Heart failure
LV: Left ventricle
LV-EF: Left ventricular ejection fraction
MR: Mitral regurgitation
NTproBNP: N-terminal pro-brain natriuretic peptide
NYHA: New York Heart Association
TMVR: Transcatheter mitral valve repair
