# Supplementary material for: “Get with the Guidelines Heart Failure Risk Score” for mortality prediction in patients undergoing MitraClip
Source: Clin Res Cardiol. 2021 Jan 31;110(12):1871–80. doi: 10.1007/s00392-021-01804-3 (PMC8639563; doi:10.1007/s00392-021-01804-3)
Supplement: Supplementary file 1 — Supplementary file1 (DOCX 20 KB) [file 392_2021_1804_MOESM1_ESM.docx]

Supplementary Table 1. Comparison of baseline characteristics between study population and patients excluded due to missing GWTG data. Displayed are median and interquartile range or numbers and percentages; p-values: Mann-Whitney-U-test or chi-square test.

|  | Study population (N=815) | Excluded patients (N=195) | p-value |
| --- | --- | --- | --- |
| Age (years) | 78 (73-83) | 78 (74-82) | 0.60 |
| Systolic blood pressure (mmHg) | 120 (110-135) | 117 (110-130) *  (N=50) | 0.17 |
| BUN (mg/dl) | 26 (18-39) | 28 (19-48) (N=66) * | 0.27 |
| Sodium (mmol/l) | 139 (137-141) | 140 (138-141) *  (N=93) | 0.49 |
| Heart rate (bpm) | 70 (64-80) | 68 (61-76) (N=65) * | 0.06 |
| Black race | 1 (0%) | 0 (0%) | 1 |
| COPD | 163 (20%) | 43 (22.4%) (N=192) * | 0.46 |
| Female, n (%) | 351 (43.1%) | 67 (34.4%) | 0.03 |
| NYHA functional class | (N=814) * | (N=194) * | 0.39 |
| I | 10 (1.2%) | 5 (2.5%) |  |
| II | 91 (11.2%) | 25 (12.9%) |  |
| III | 572 (70.3%) | 133 (68.6%) |  |
| IV | 141 (17.3%) | 31 (16%) |  |
| Left ventricular ejection fraction | (N=812) * | (N=187) * | 0.64 |
| <30%, n (%) | 185 (22.8%) | 46 (24.6%) |  |
| 30–50%, n (%) | 275 (33.9%) | 67 (35.8%) |  |
| >50%, n (%) | 352 (43.3%) | 74 (39.6%) |  |
| Secondary etiology of MR, n (%) | 509 (62.5%) | 101 (53.2%) *  (N=190) | 0.02 |
| Peripheral arterial disease, n (%) | 115 (14.1%) *  (N=814) | 37 (19.8%) *  (N=187) | 0.052 |
| Previous myocardial infarction, n (%) | 233 (28.7%) *  (N=812) | 67 (35.8%) *  (N=187) | 0.055 |
| Previous TIA, n (%) | 8 (1%) | 15 (7.9%) (N=191) * | <0.001 |
| Previous stroke, n (%) | 101 (12.4%) | 7 (3.7%) (N=191) * | <0.001 |
| Atrial fibrillation, n (%) | 511 (63.2%) *  (N=809) | 133 (69.6%) *  (N=191) | 0.09 |
| Hypertension, n (%) | 670 (82.2%) | 170 (89%) *  (N=191) | 0.02 |
| Diabetes mellitus, n (%) | 255 (31.3%) | 44 (23%) *  (N=191) | 0.03 |
| Estimated GFR (ml/min/1.73m^2^) | 48 (35-64) *  (Ν=806) | 48 (35-62) *  (Ν=172) | 0.33 |
| Previous CABG, n (%) | 344 (42.2 %) | 61 (31.9%) *  (N=191) | 0.01 |
| NTproBNP (ng/l) | 2,529 (1,392-5,499) *  (Ν=637) | 3,304 (1,231-7,657) *  (Ν=129) | 0.22 |
| Euroscore (%) | 19.2 (10.4-31.3) *  (Ν=791) | 12.9 (7.4-24.2) *  (Ν=190) | <0.001 |

*only available in N of the patients

Supplementary Table 2: Comparison of observed and expected deaths events by GWTG-HF score quartiles.

| Score quartile | Observations | Observed deaths | Expected deaths |
| --- | --- | --- | --- |
| 1 | 210 | 19 | 22.4 |
| 2 | 218 | 34 | 35.9 |
| 3 | 186 | 47 | 38.8 |
| 4 | 201 | 77 | 79.8 |

Supplementary Table 3: Bivariate Cox regression analysis for mortality. Included are the GWTG-HF risk score and the Euroscore.

|  | Hazard ratio | 95%-Confidence interval | p-value |
| --- | --- | --- | --- |
| GWTG-HF | 1.08 | 1.06-1.10 | <0.001 |
| Euroscore | 1.01 | 1.0-1.02 | 0.013 |

Abbreviations for Tables:

bpm: beats per minute; BUN: blood urea nitrogen; CABG: coronary artery bypass grafting; COPD: chronic obstructive pulmonary disease; LV-EF: left ventricular ejection fraction; eGFR: estimated glomerular filtration rate; GWTG-HF: Get-with-the-guidelines heart failure; IQR: interquartile range; MR: mitral regurgitation; NTproBNP: N-terminal pro-brain natriuretic peptide; NYHA: New York Heart Association; TIA: transitory ischemic attack

Supplementary Figure 1: Arjas like plots comparing observed and expected event counts of death at each event time by GWTG-HF score quartiles.

Supplementary Figure 2. Kaplan Meier plot for mortality by GWTG-HF score tertiles. Subgroup analysis for primary mitral regurgitation.

Logrank p<0.001

Days

No. at risk

GWTG-HF 0-39

98 97 96 93 91 90

GWTG-HF 40-44

97 90 88 86 85 82

GWTG-HF 45+

111 90 89 82 80 78

Supplementary Figure 3. Kaplan Meier plot for mortality by GWTG-HF score quartiles. Subgroup analysis for secondary mitral regurgitation.

Logrank p<0.001

Days

No. at risk

GWTG-HF 0-37

136 134 131 130 129 128 GWTG-HF 38-42

135 128 123 121 117 116

GWTG-HF 43-46

113 101 95 88 84 83

GWTG-HF 47+

125 105 98 93 85 82

Supplementary Figure 4. Kaplan Meier plot for mortality by GWTG-HF score quartiles. Subgroup analysis for ejection fraction <50%.

Logrank p<0.001

Days

No. at risk

GWTG-HF 0-37

106 103 102 101 100 99 GWTG-HF 38-42

120 114 110 108 104 102

GWTG-HF 43-46

113 98 93 90 89 89

GWTG-HF 47+

124 106 101 92 85 83

Supplementary Figure 5. Kaplan Meier plot for mortality by GWTG-HF score quartiles. Subgroup analysis for ejection fraction ≥50%.

Logrank p<0.001

Days

No. at risk

GWTG-HF 0-37

104 103 100 98 97 97 GWTG-HF 38-42

98 94 91 90 89 96

GWTG-HF 43-46

73 64 63 57 54 52

GWTG-HF 47+

77 62 59 56 54 52

Supplementary Figure 6. Comparison of area under the ROC curve for GWTG-HF score and Euroscore.
