# Supplementary material for: “Get with the Guidelines Heart Failure Risk Score” for mortality prediction in patients undergoing MitraClip
Source: Clin Res Cardiol. 2021 Jan 31;110(12):1871–80. doi: 10.1007/s00392-021-01804-3 (PMC8639563; doi:10.1007/s00392-021-01804-3)

supplementary figure 1

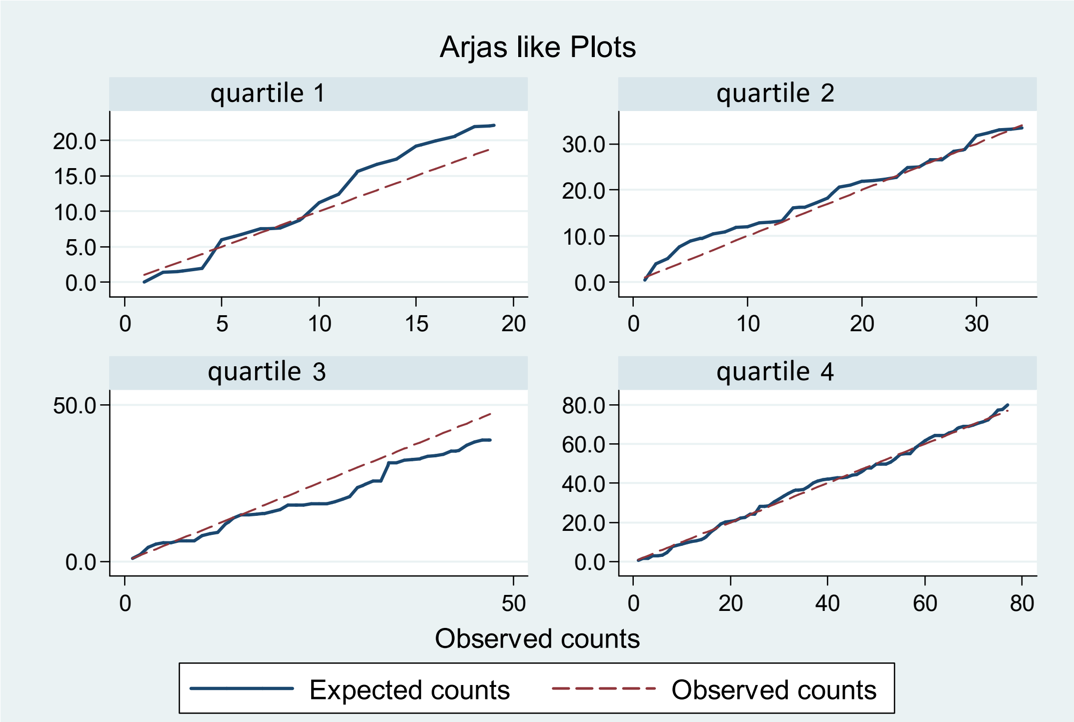

supplementary figure 2

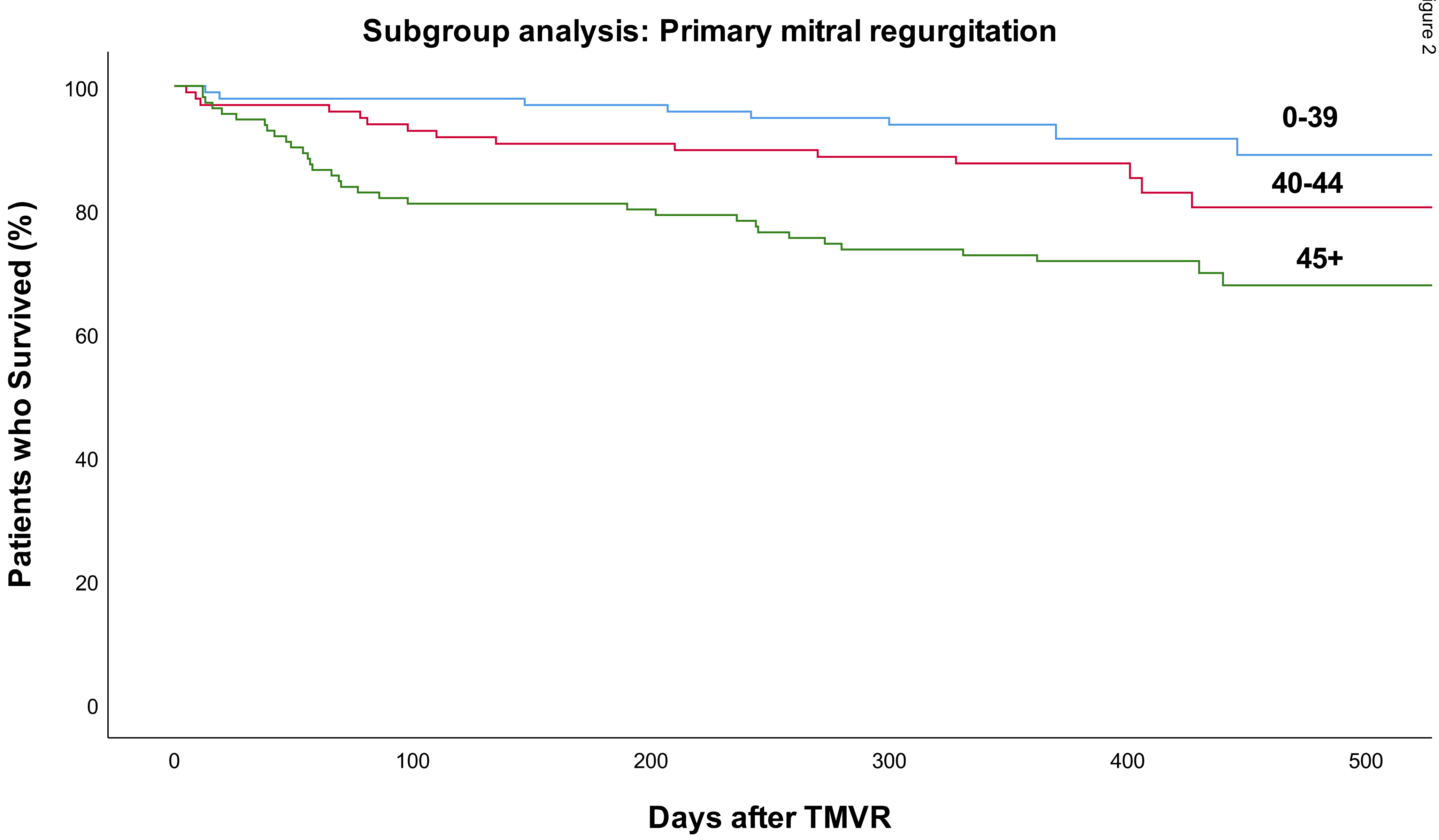

supplementary figure 3

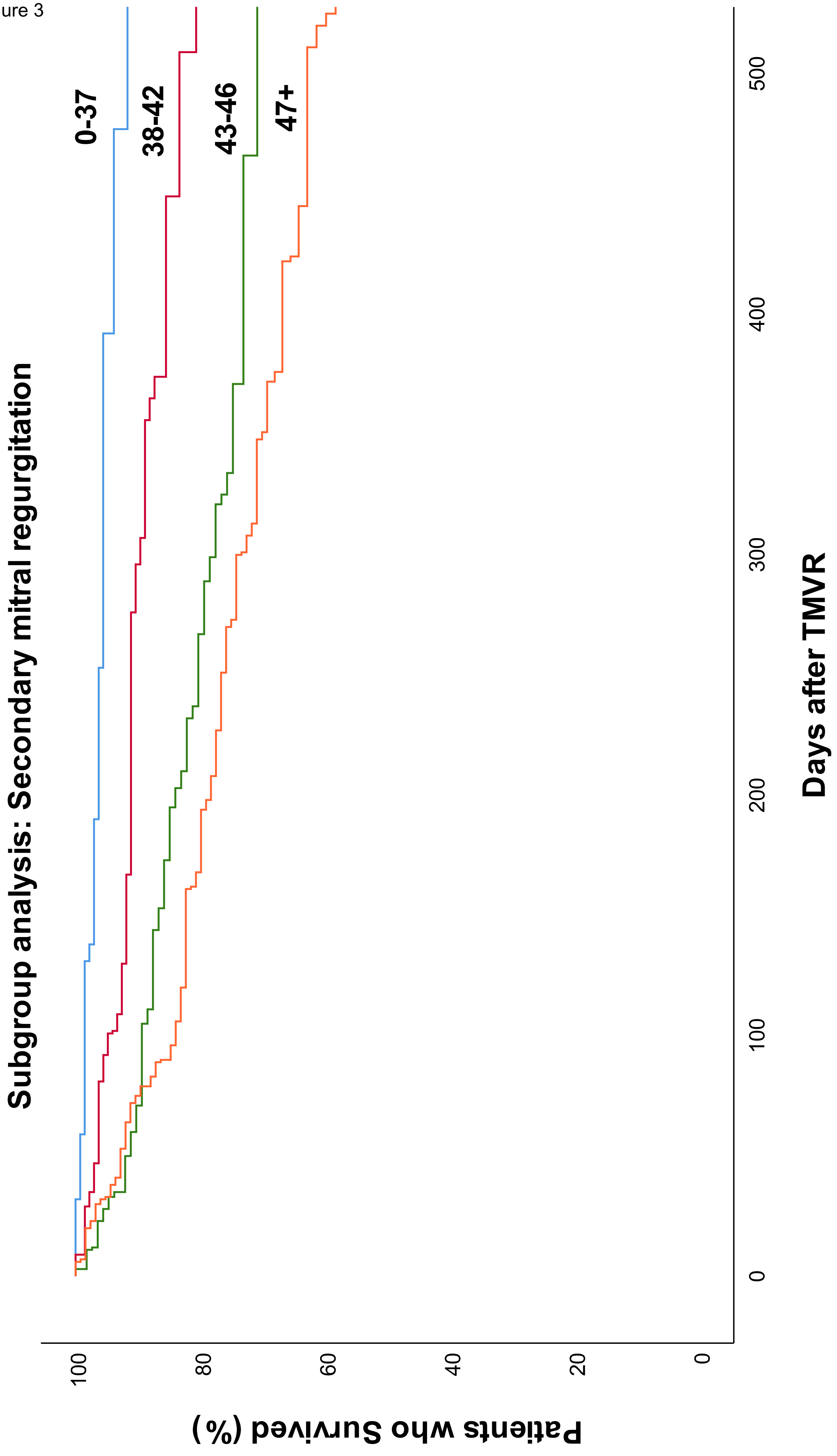

supplementary figure 4

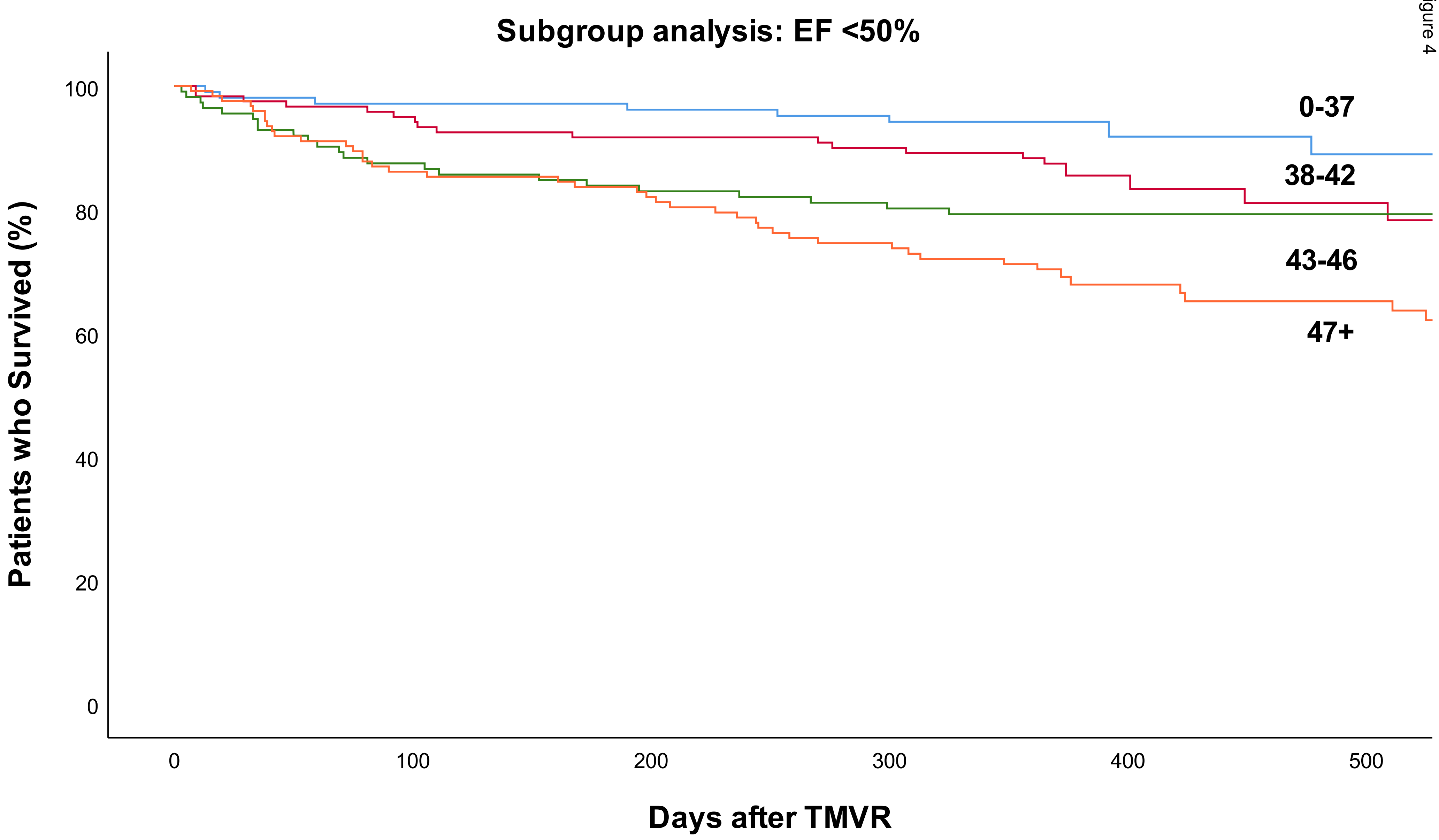

supplementary figure 5

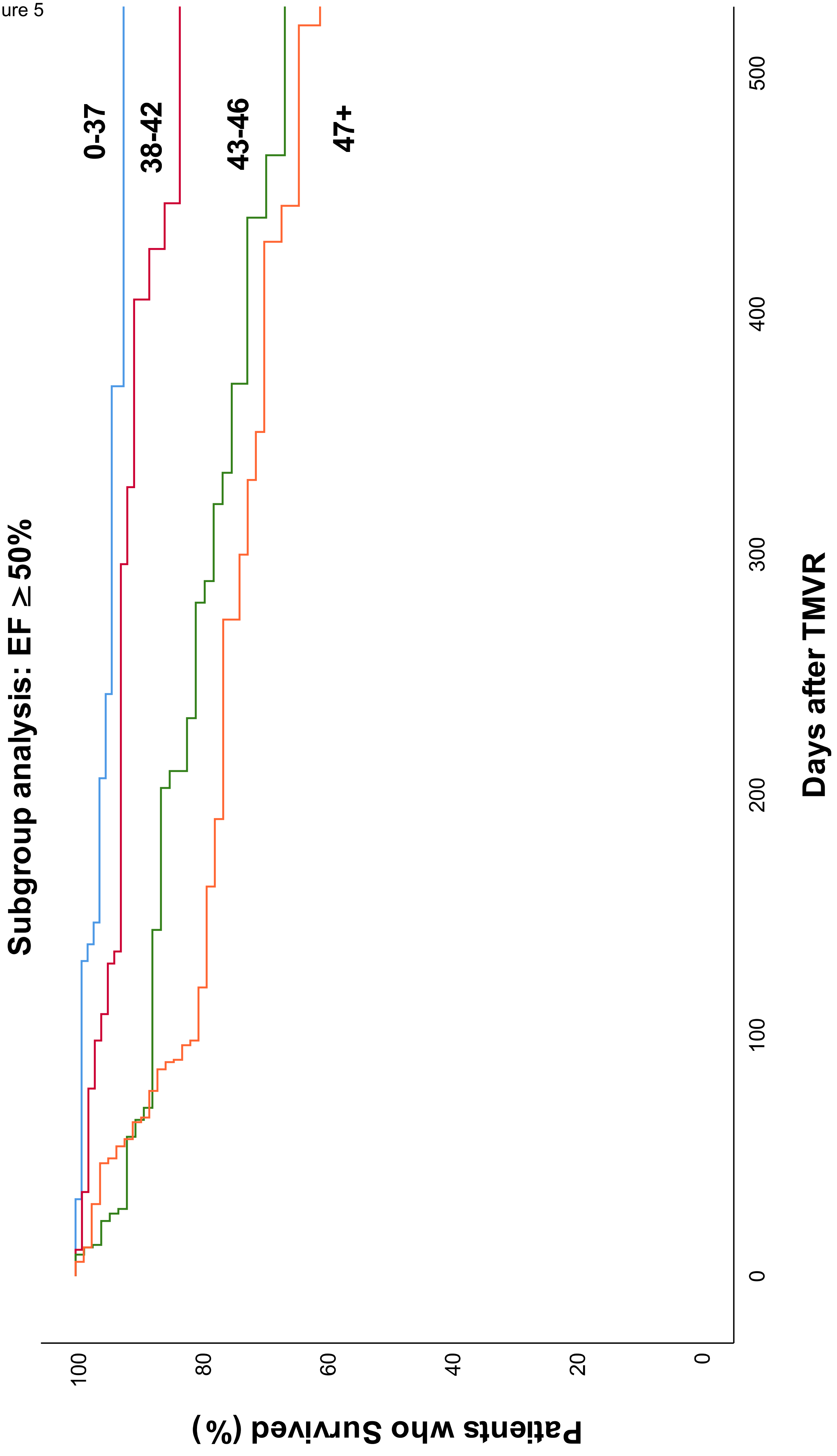

supplementary figure 6

Comparison of area under the ROC curve for GWTG-HF score and Euroscore

ROC analysis for 30-day mortality

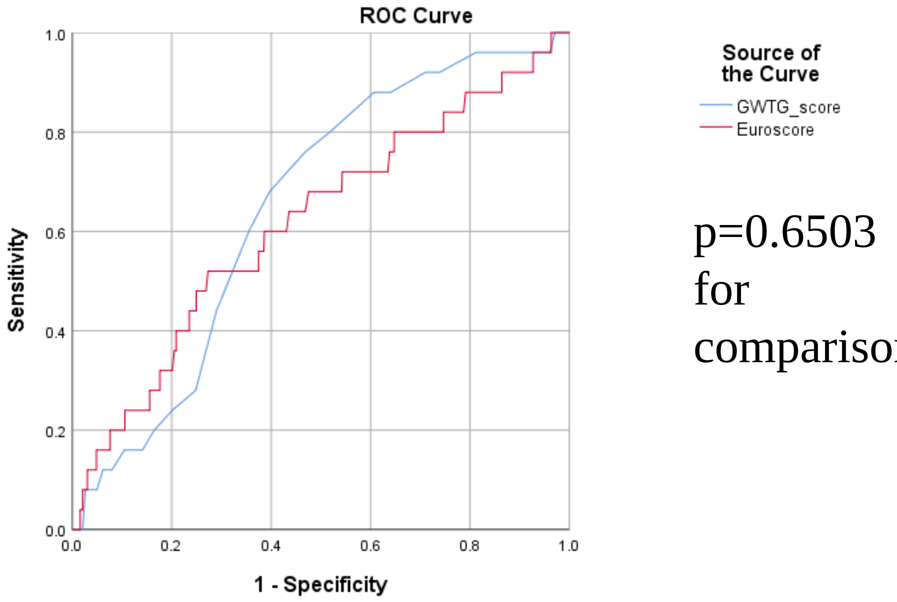

p=0.6503 for comparison

	Area Under the Curve	p-value	95%-Confidence Interval
GWTG-HF	0.649	0.011	0.58-0.74
Euroscore	0.617	0.046	0.50-0.74

ROC analysis for 1-year mortality

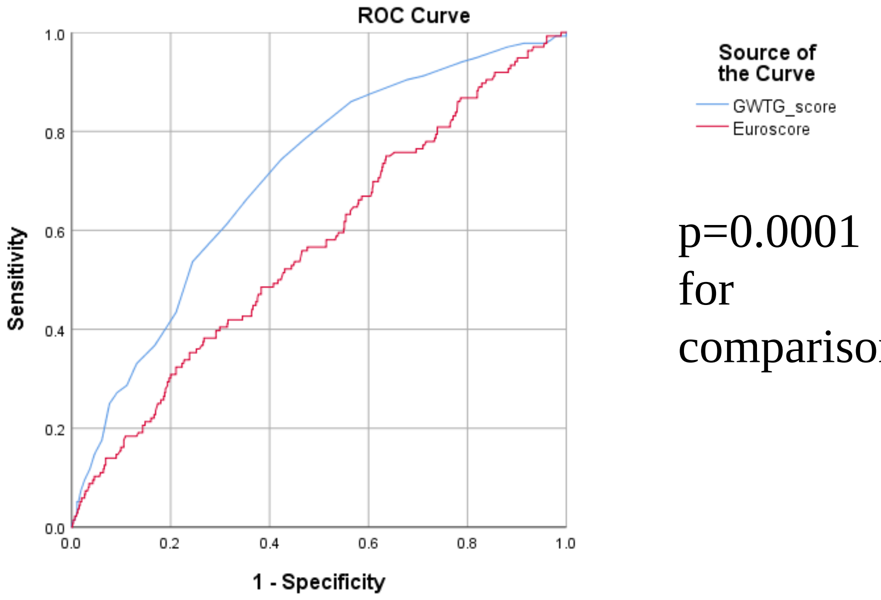

p=0.0001 for comparison

	Area Under the Curve	p-value	95%-Confidence Interval
GWTG-HF	0.706	<0.001	0.66-0.75
Euroscore	0.568	0.013	0.52-0.62

graphic abstract

“Get with the Guidelines Heart Failure Risk Score” for Mortality Prediction in Patients Undergoing MitraClip

815 patients undergoing MitraClip in the University hospitals of Bonn, Cologne and Duesseldorf

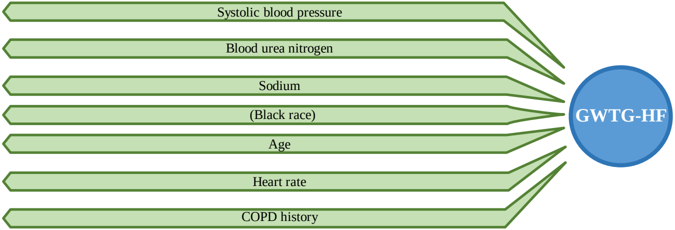

In-hospital and 1-year mortality according to GWTG-HF risk score quartiles

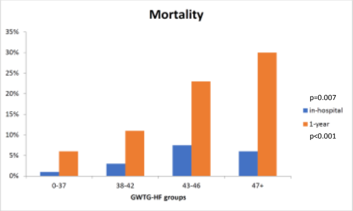

Frequency distribution of the GWTG-HF risk score and associated hazard ratio for mortality

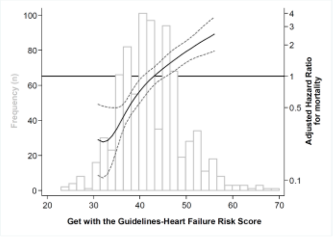

Supplement: Supplementary file 2 — Supplementary file2 (PDF 860 KB) [file 392_2021_1804_MOESM2_ESM.pdf]
